# Supplementation of Thymoquinone Nanoparticles to Semen Extender Boosts Cryotolerance and Fertilizing Ability of Buffalo Bull Spermatozoa

**DOI:** 10.3390/ani13182973

**Published:** 2023-09-20

**Authors:** Wael A. Khalil, Mahmoud A. E. Hassan, Mostafa A. El-Harairy, Sameh A. Abdelnour

**Affiliations:** 1 Department of Animal Production, Faculty of Agriculture, Mansoura University, Mansoura 35516, Egypt; 2 Animal Production Research Institute, Agriculture Research Centre, Ministry of Agriculture, Giza 12619, Egypt; m.hassan55213@gmail.com; 3 Department of Animal Production, Faculty of Agriculture, Zagazig University, Zagazig 44511, Egypt

**Keywords:** cryopreserved semen, buffalo, sperm quality and kinematics, apoptotic, acrosome exocytosis, thymoquinone nanoparticle

## Abstract

**Simple Summary:**

Semen cryopreservation is a reliable technique used in artificial reproduction technology to preserve and transmit buffaloes’ superior genetic qualities. Buffalo sperm cells are highly susceptible to cryopreservation due to the abundant polyunsaturated fatty acids in their plasma membrane. The use of nanoparticles in freezing extenders is one of the most recent techniques for reducing the detrimental effects of cryopreservation on the post-thawed quality of buffalo spermatozoa. In this study, thymoquinone nanoparticles (TQNPs) were used for the first time as an effective method to enhance the sperm quality and fertilizing ability of frozen–thawed buffalo sperm. The addition of optimal levels of TQNPs (25 to 50 µg/mL) improved post-thawed sperm parameters and sperm kinematics, as well as maintained the morphology of sperm by supporting the total antioxidant capacity and reducing membrane damage, apoptotic sperm, and oxidative stress-related markers. These data can provide a novel nanotechnology-based strategy for enhancing the cryopreservation efficiency of buffalo sperm.

**Abstract:**

Thymoquinone nanoparticles (TQNPs) are broadly utilized in numerous pharmaceutical applications. In the present study, we tested the effects of TQNP supplementation on sperm quality and kinematics, acrosome exocytosis, oxidative biomarkers, apoptosis-like and morphological changes of frozen–thawed buffalo sperm, as well as the fertilizing capacity. Semen was collected from buffalo bulls, diluted (1:10; semen/extender), and divided into five aliquots comprising various concentrations of TQNP 0 (CON), 12.5 (TQNP12.5), 25 (TQNP25), 37.5 (TQNP37.5), and 50 (TQNP50) µg/mL, and then cryopreserved and stored in liquid nitrogen (−196 °C). The results revealed that TQNPs (25 to 50 µg/mL) provided the most optimal results in terms of membrane integrity (*p <* 0.001) and progressive motility (*p <* 0.01). In contrast, TQNP50 resulted in a greater post-thawed sperm viability (*p* = 0.02) compared with other groups. The addition of TQNPs to the extender had no discernible effects on sperm morphology measures. Sperm kinematic motion was significantly improved in the TQNP50 group compared to the control group (*p* < 0.01). TQNPs effectively reduced the content of H_2_O_2_ and MDA levels and improved the total antioxidant capacity of post-thawed extended semen (*p <* 0.01). The addition of TQNP significantly increased the number of intact acrosomes (*p <* 0.0001) and decreased the number of exocytosed acrosomes (*p <* 0.0001). A significant reduction in apoptosis-like changes was observed in TQNP groups. The non-return rates of buffalo cows inseminated with TQNP50-treated spermatozoa were higher than those in the control group (*p <* 0.05; 88% vs. 72%). These findings suggested that the freezing extender supplemented with TQNPs could effectively enhance the cryotolerance and fertility of buffalo sperm.

## 1. Introduction

Globally, water buffalo (*Bubalus bubalis*) is an imperative livestock species, providing meat, milk, skin, and draught power. Despite being well established in some parts of the world, water buffaloes display an exceptional set of challenges owing to their late sexual maturity, long generation intervals, and silent heat, which impedes genetic improvement [1,2]. For enhancing the reproductive capacity of water buffaloes, assisted reproductive technologies (ARTs) such as in vitro maturation (IVM), artificial insemination (AI), in vitro fertilization (IVF), cloning, and nuclear transfer have been utilized worldwide [1,3,4]. Semen cryopreservation is a crucial procedure for ART [5,6]. The primary objectives of cryopreservation are to maximize the number of viable sperm after thawing and to preserve their original quality parameters, such as motility, viability, integrity of the plasma membrane and acrosome, DNA integrity, as well as biological functions associated with the fertilizing ability [5]. While semen cryopreservation is a well-established process applied in AI, up to 50% of the spermatozoa cannot survive after the freeze–thaw process [1,5].

Cold shock, osmotic stress, intracellular ice crystal formation, extreme reactive oxygen species (ROS) release, and fluctuations in antioxidant protection systems can all lead to cryo-injury [7,8]. The freeze–thaw process substantially increases ROS levels, creating an imbalance with the antioxidant capacity of the sperm and resulting in oxidative stress (OS) [9]. This leads to sperm dysfunction and low fertilizing capacity. Buffalo sperm is sensitive to cryo-storage events due to high levels of polyunsaturated fatty acids (PUFAs) in its plasma membrane. Therefore, it is more susceptible to OS than bovine sperm when stored at low temperatures or during cryopreservation [5]. This finding may explain why the fertilization potential of buffalo sperm decreases following a freeze–thaw process. While ROS such as hydrogen peroxide, superoxide anions, and nitric oxide play important roles in cell signaling, excessive levels can induce sperm dysfunction, leading to male infertility [8,10,11]. Consequently, improving sperm quality or functionality by limiting OS and strengthening the antioxidant defense system in cryopreserved semen via extender supplements with natural antioxidants [9,12,13,14] is an applicable strategy to improve the cryo-resistance and fertilizing ability of buffalo sperm.

The strategy of the use of antioxidants to boost semen freezability and increase cryopreservation due to antioxidant and anti-apoptotic molecules is increasing. Black cumin (*Nigella sativa*) is a common vegetable plant with multiple therapeutic effects [15,16,17]. Thymoquinone (TQ) is the main component of *Nigella sativa* and accounts for 30–40% of its oil, with several significant biological activities. Numerous pharmacological properties of TQ have been discovered, including anti-inflammatory, immunomodulatory, antioxidant, anticancer, and anti-asthmatic activities [18,19,20,21,22]. The drawback of considering TQ as a principal therapeutic drug is interrelated with an inferior bioavailability and significant hydrophobic properties, resulting in low solubility.

The application of nanotechnology to herbal medicine is one of the most rapidly expanding fields [23]. Moreover, the application of nanotechnology has numerous benefits that have contributed to an increase in the use of herbal medication, e.g., in the treatment of various cancers and chronic syndromes [24]. In the past five years, our research team has achieved promising results for sperm functionality and quality after the addition of nanoparticles to freezing extenders such as nano-curcumin [25], nano-selenium [26], nano-essential oils [27], and alpha-lipoic-acid-loaded nanoliposomes [5] in rabbit, bull, goat, and buffalo, respectively. The preparation of nanoparticles from natural sources could improve their bioavailability, effectiveness, and cryopreserved sperm quality in various animal species [23,24,28,29]. The thymoquinone nanoparticle (TQNP) has been extensively used in therapeutic therapy [28]. Several authors have demonstrated the favorable impacts of *Nigella sativa* extract or their oil in improving sperm quality during cryopreservation in different animals [13,15,16,17,30]. In addition, the free TQ improved post-thawed sperm in rams [22], and protected sperm functionality from various damages [18,31,32]. However, the fact that the nanoparticle formulation of TQ (TQNP) has numerous potential scientific applications, their role in enhancing sperm cryotolerance and fertilizing ability remains unexplored. There is still no specific cryopreservation method for water buffalo, and a significant percentage of frozen–thawed sperm does not meet the quality requirements for AI. Therefore, exploring the use of a special semen-freezing extender supplemented with TQNPs may be appropriate for ensuring an optimal breeding platform in Egyptian buffalo. In the current research, the effects of TQNPs on semen quality, sperm kinematics, acrosome exocytosis, oxidative biomarkers, apoptosis-like changes, the ultrastructure of frozen–thawed buffalo sperm, and fertilizing ability were examined. The results will aid in improving sperm cryopreservation techniques for Egyptian buffaloes.

## 2. Materials and Methods

### 2.1. Ethical Statement

The current research was conducted at Mahalet Mussa Station, Sakha, Kafr El-Sheikh Governorate, Egypt. The animal use and handling procedures were approved by the Institutional Animal Use and Care Committee (IACUC) at Zagazig University, Egypt (ZU IACUC/2/F/173/2022).

### 2.2. Preparation of Thymoquinone Nanoparticles (TQNPs)

As previously indicated by Hassan et al. [5], the traditional fine-film hydration procedure was monitored during the TQNP preparation. Accurately weighed quantities of soybean lecithin (30 mg) and thymoquinone (5 mg) were dissolved in 10 mL of a 1:2 mixture of methanol and chloroform. The mixture was evaporated into a transparent fine film in a vacuum system equipped with a rotary evaporator at 60 °C (Heidolph LABOROTA 4000, Serial No. 030002422, Wansdorf, Germany). After complete evaporation, the dried tinny lipid flick was hydrated with DW (deionized water, 10 mL) at 60 °C for 20 min and 120 rpm to obtain a crude suspension. Consequently, the suspension was isolated through sonication for 20 min using an ultrasonic bath (Sonix, New York, NY, USA, SS101H230). Furthermore, additional standardization was performed utilizing an ultrasonic probe (Serial No. 2013020605, Model CV 334) with a homogenizer (VC 505, USA) in an ice bath under the following conditions to produce one-phase nanoparticles: timer set to 3 min, amplitude of 60%, and pulser of 1 s ON/1 s OFF.

### 2.3. Determination of Thymoquinone Nanoparticles Features

TQNP average vesicular sizes (Z-average), surface charge, and polydispersity index (PDI) were determined using a Zetasizer Nano ZS analyzer (Malvern Instruments, Malvern, UK) and the dynamic light scattering (DLS) technique. After adequate dilution with DW at 25 °C, the freshly prepared formula was tested in triplicate. The TQNP surface charge, expressed as zeta potential (ZP), which detects the vesicle’s electrophoretic mobility in an electric field, was measured in triplicate using the same apparatus and DW dilution as described in the previously mentioned Z-average measurement.

The morphology of the freshly synthesized TQNPs was imaged using transmission electron microscope (TEM), (JEOL JEM-2100, Tokyo, Japan) at 200 kV. After a suitable extension with DW, the sample was loaded onto a carbon-coated copper grid and observed using TEM. Digital micrograph and soft imaging viewer software (Gatan Microscopy Suite Software, version 2.11.1404.0) were utilized for image capture, analysis, and particle measurement.

### 2.4. Animal Management and Semen Collection

Five proven fertility Egyptian buffalo bulls aged 4–6 years acquired at Mahalet Mussa Station, Sakha Kafr El-Sheikh Governorate, Egypt, were used in the current study. All bulls were housed under standard management conditions. Semen samples were obtained once weekly from the five buffalo bulls for seven successive weeks (n = 35 total ejaculates) using artificial vaginas and maintained at 42–45 °C. Two qualified researchers examined each ejaculate for progressive motility using a phase contrast microscope (100×) as soon as the sperm had been collected. In the freezing experiment, only ejaculates with acceptable progressive motility (≥75%), viability (≥80%), abnormality (≤15%), and sperm concentration (≥8 × 10^8^/mL) were selected, pooled, and used.

### 2.5. Extender Preparation and Experimental Design

Before buffalo semen collection, the extender was prepared by dissolving citric acid 1.675 g, Tris 3.028 g, glycerol 6.0 mL, fructose 1.25 g, and egg yolk 20 mL in double-distilled water (DDW, up to 100 mL) [26]. Furthermore, streptomycin (100 µg/mL) and penicillin (100 IU/mL) were also added. For each replicate, the extender was aliquoted into five 15 mL test tubes and stored in a water bath at 37 °C. The first test tube without any additions was designated as control (CON), while extenders in the other tubes were supplemented with different concentrations of TQNPs as follows, 12.5, 25, 37.5, and 50 µg/mL, designated as groups TQNP12.5, TQNP25, TQNP37.5, and TQNP50, respectively. The extension frequency of the semen with extenders was 1:10 (sperm/extender), yielding an initial dose of 80 × 10^6^ spermatozoa/mL in the diluted semen.

### 2.6. Freezing and Thawing

Extended semen was gently shaken and placed in a water bath (37 °C) and equilibrated at 5 °C for 4 h (equilibration stage) before packing into French straws (0.25 mL, IVM technologies, Marseille, France). The straws were suspended at 4 cm above the surface of liquid nitrogen for 10 min, and then submerged in liquid nitrogen. The semen was cryopreserved in liquid nitrogen for one month, and then thawed at 37 °C for 30 s in a water bath for further semen assessments.

### 2.7. Assessments of Frozen–Thawed Semen Features

After one month of cryopreservation, frozen straws of each treatment were thawed for 30 s in a 37 °C water bath [10]. The percentage of progressive sperm motility, defined as the sperm’s ability to move forward in a long semi-arc pattern, was determined using a phase-contrast microscope (DM 500, Leica, St. Gallen, Switzerland) equipped with a warm stage set at 37 °C at a magnification of 100×. For the analysis, a 10 µL sample of diluted semen was placed on a prewarmed slide and covered with a coverslip. The analysis was carried out by the same skilled investigator, blindly, and repeated three times for each sample [26].

**Figure 1:**
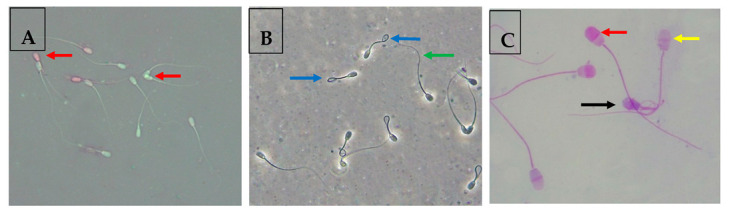
(**A**) Assessment of buffalo sperm viability using the eosin/nigrosine test. Red arrows indicate viable sperm cells appeared white-stained, and non-viable sperm cells were pink-stained. (**B**) Assessment of plasma membrane integrity of buffalo sperm cells using the HOST. Blue arrows highlight sperm cells with an intact plasma membrane, and green arrows highlight sperm cells with a damaged plasma membrane. (**C**) Assessment of acrosome integrity of buffalo sperm cells using the Giemsa staining. This test leads to differentiation of three sperm cell populations: viable sperm with an intact acrosome (red arrow), viable sperm with an exocytosed acrosome (yellow arrow), and non-viable sperm with an exocytosed acrosome (black arrow).

The sperm viability was assessed using the eosin/nigrosine stain method according to Moskovtsev and Librach [33] protocol. For preparing the eosin/nigrosin solution, 0.67 g of eosin and 0.9 g of sodium chloride were dissolved in distilled water (100 mL). The mixture was heated gently, followed by adding 10.0 g of nigrosine and kept under dark conditions until use. Samples (10 µL) from each group were incubated with the eosin/nigrosin solution (10 µL) for 2 min at 25 °C and then smeared on hot glass slides for air-drying. At 400× magnification, at least 200 sperm cells were observed under a light microscope. For microscope examination, unstained sperm cells were considered viable, while pink-stained sperm were considered dead (Figure 1A).

In the same scope, the number of sperm cells with abnormal tail morphology (coiled tail, broken tail, terminally coiled tail, double tail) and head morphology (microcephalic head, pear-shaped head, round short head, loose head, double head) was also recorded (as percentages), as previously described [34].

For assessing the membrane integrity (%) ratio, a hypo-osmotic swelling (HOST) test, as described by the protocol in [26], was performed. In addition, this method was mentioned in detail in our previous works [5,26]. A total of 200 sperm cells were estimated for their swelling capability in HOST. Sperm cells with a swollen or coiled tail were considered to have an intact plasma membrane (Figure 1B).

### 2.8. Computer-Assisted Sperm Analysis

According to the method of Dessouki et al. [35], the software CASA sperm analyzer (Sperm Vision; Ref: 12520/5000; Minitube Hauptstrae 41. 84184 Tiefenbach, Germany) was applied to provide more information on the live images of various sperm motion characteristics captured by the Olympus computer-assisted microscope. The microscope Olympus BX (Hamburg, Germany) was connected with a rapid scan digital camera to image (60 frames per second) and capture at 60 Hz under ×4 dark-field illumination. This system was set up at 37 °C. In each treatment, approximately 1500 spermatozoa were analyzed using CASA. The motion characterization of sperm was documented, counting distance curved line (DCL, µm), distance straight line (DSL, µm), velocity average path (VAP, µm/s), distance average path (DAP, µm), velocity curved line (VCL, µm/s), linearity (LIN = VSL/VCL), wobble (WOB = VAP/VCL), straightness (STR = VSL/VAP), beat cross frequency (BCF, Hz), and amplitude of lateral head displacement (ALH, µm).

### 2.9. Assessment of Acrosome Integrity

The sperm acrosome integrity rate was assessed based on the Giemsa Staining technique. Frozen–thawed samples (200 μL) were placed into a plastic tube containing the same volume of 0.2% trypan blue and then incubated for 10 min at 37 °C in a water bath. Sperm were extended with modified Brackett and Oliphant medium without albumin (2 mL) and centrifuged for 6 min at 700 g based on the protocol [36]. The supernatant was discarded, and the pellet’s spermatozoa were re-suspended in 1–2 mL of the same medium and centrifuged. This phase was repeated until the suspension was either pure or light blue. A 10–20 L aliquot of the sperm suspension was placed on a glass slide and smeared using a second glass slide. The slides were rapidly dried on a 40 °C heating stage. Sperm on the slides were successively stained for one hour with a freshly prepared 10% Giemsa stock solution in distilled water.

Following staining, the slides were washed under a stream of distilled water and air-dried. To determine the acrosome integrity under bright-field microscopy, about 100 sperm cells were randomly selected per slide and examined as follows (Figure 1C):

- Viable sperm with an intact acrosome: The acrosomal region was stained in pink/purple, and the post-acrosomal region appeared white-stained. Viable sperm with exocytosed acrosome: post-acrosomal region was white, and acrosome was white (true acrosome exocytosis).
- Non-viable sperm with an intact acrosome: the acrosomal region stained either dark pink or purple, and the post-acrosomal region stained either blue or dark blue.
- Non-viable sperm with a reacted acrosome: the acrosomal region stained either in white or gray and the post-acrosomal region stained in blue (false acrosome exocytosis).

### 2.10. Assessment of Apoptosis through Flow Cytometry

A flow cytometry instrument was utilized to evaluate apoptosis-like changes in frozen–thawed spermatozoa using Annexin V staining [37]. Each group’s extended sperm was centrifuged, and the isolated sperm cells were suspended in 2 mL of binding buffer and thoroughly mixed. In addition, the suspension (100 µL) was mixed in PI (5 μL; phycoerythrin label) and Annexin V (5 μL; fluorescein isothiocyanate label), incubated in dark conditions for at least 15 min, and then suspended in 200 μL of binding buffer. For achievement and evaluation of the apoptotic sperm status, flow cytometry examination using an Accuri C6 Cytometer (BD Biosciences, San Jose, CA, USA) and software (Becton Dickinson) was achieved [38]. The percentages of positive or negative Annexin V (A−/A+), PI (PI−/PI+), and the double-positive cells were assessed.

According to Peña et al. [39], sperm were categorized into four classes:

(a) Viable cells: without fluorescence signal and membrane dysfunction (A−/PI−).
(b) Apoptotic sperm cells: viable cells labeled with Annexin V but without PI (A+/PI−).
(c) Necrotic sperm cells: non-viable cells labeled with PI without Annexin V and with complete membrane loss (A−/PI+).
(d) Necrotic sperm cells: Non-viable cells labeled with Annexin V and PI and with damaged permeable membranes (A+/PI+). Lastly, the number of spermatozoa in each previous category was recorded in each group.

### 2.11. Ultrastructure Assay of the Cryopreserved Spermatozoa

The ultrastructural modifications of buffalo-bull sperm cells were investigated using TEM according to the protocol of Khalil et al. [26], with some minor modifications. Frozen–thawed semen was fixed with 2% glutaraldehyde in PBS for 2–3 h, and then washed three times in PBS via centrifugation for 5 min at 4 °C. Finally, the mixture was incubated in 1% of osmium tetroxide for approximately 2 h at 4 °C. Fixed spermatozoa were dehydrated in acetone and embedded in Epon resin. Ultrathin sections of sperm samples were cut, expending the RMC ultra-microtome, and stained with lead citrate and uranyl acetate. Randomly, fields were examined using a TEM (JEOL JEM-2100, Tokyo, Japan) equipped with an AMT, Optronics CCD camera.

### 2.12. Oxidative Biomarkers Assays

Frozen–thawed semen samples were centrifuged for 10 min at 6000 rpm, and then the extender was separated and stored at −20 °C. The concentrations of TAC (total antioxidant capacity; TA 2513), MDA (Malondialdehyde; MD 2529), H_2_O_2_ (hydrogen peroxide, HP25), and NO (nitric oxide, NO2533) were measured in extender according to the methods of [40,41,42,43], at the wavelengths 505, 532, 510, and 540 nm, respectively. The linearity indices for TAC, MDA, H_2_O_2_, and NO were up to 2 mM/L, 100 nmol/mL, 1.5 mM/L, and 200 µmol/L, respectively. All tests were conducted using a spectrophotometer (Spectro UV-Vis Auto, UV-2602; Culver City, CA, USA), and the commercial kits were obtained from Biodiagnostic Company (Giza, Egypt) and utilized in accordance with the manufacturer’s guidelines.

### 2.13. Fertility Rate

A total of 100 multiparous buffalo cows exhibiting spontaneous estrus were arbitrarily selected and divided into two treatment groups (1st treatment was the control group, and 2nd treatment was TQNP 50 µg/mL, with the optimal treatment based on the in vitro outcome indicators). Each group (50 females) was artificially inseminated with frozen/thawed semen. A single AI technician performed all inseminations. At 56 days of artificial insemination, the number of cows with a non-return rate (56-NRR) was recorded.

### 2.14. Statistical Analysis

Data were analyzed using Microsoft Excel version 16 (Microsoft Corporation, Redmond, WA, USA). A Shapiro–Wilk test was conducted to check for normality, as described by Razali and Wah [44]. The significant effects of the treatments were examined according to the one-way ANOVA (PROC ANOVA; SAS Institute Inc., Madison, WI, USA, 2012). The level of statistical significance was set at α = 0.05. The following mathematical model was applied to analyze all measurements, Yij = μ + TRTi + eij, where Yij = observations, μ = overall mean, TRT = effect of the TQNB (i, 1 to 5), and eij = random error. Results are expressed as mean ± SE. Tukey’s test was used to perform pairwise comparisons between means in case a significant effect was detected. The association between the TQNP50 group together with the control and the 56-day non-return rates was examined using the chi-square test (χ^2^). Statistical significance between means was set at a *p*-value less than 0.05.

## 3. Results

**Figure 2:**
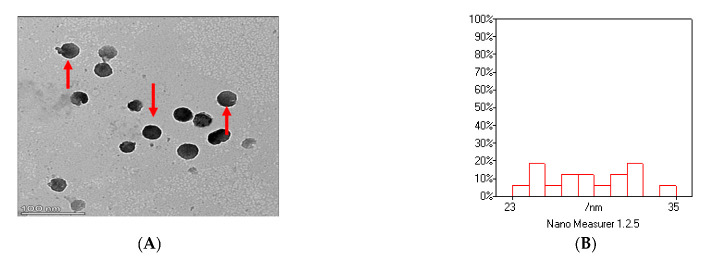

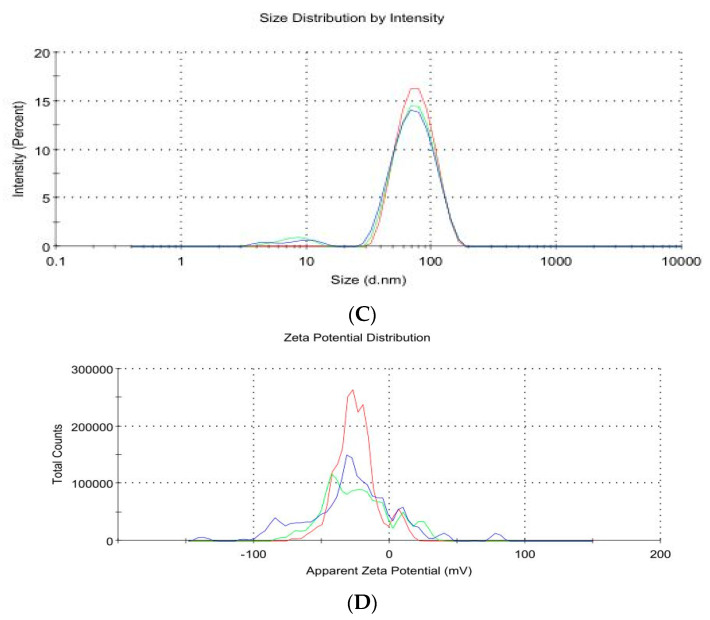
The characterization of TQNPs through TEM (**A**) shows a spherical morphology (red arrows) with a good size distribution (**B**). The nanoscale of TQNPs (**C**), and the zeta potential distribution of TQNPs is −32.5 (**D**).

As shown in Figure 2A–D, the image of thymoquinone nanoparticles (TQNPs) had a spherical morphology (Figure 2A) with a good size distribution (Figure 2B). Figure 2C shows the nanoscale of TQNPs. Moreover, TQNPs had a nanoscale size of around 35 nm, a spherical shape, and a good dispersity that agreed with the zeta potential distribution of −32.5 m/V (Figure 2D).

### 3.1. Effects of TQNP on Buffalo Bull Semen after Equilibration (5 °C for 4 h)

As depicted in Table 1, the groups TQNP25, TQNP37.5, and TQNP50 had higher progressive motility (*p* < 0.004), viability (*p* < 0.001), membrane integrity (*p* < 0.001), and sperm morphology values (*p* < 0.009) than the CON group after equilibration (5 °C for 4 h). Adding TQNPs (25–50 µg/mL) to the extender did not significantly affect progressive motility, membrane integrity, or viability among the same groups. A slight improvement was detected in all sperm variables after equilibration (5 °C for 4 h) in TQNP12.5. However, the difference was not statistically significant in the CON group. In addition, all TQNP groups significantly decreased the sperm morphology compared with the CON group (*p* < 0.001).

### 3.2. Effects of TQNPs on Post-Thawed Buffalo Bull Semen

According to Table 2, the progressive motility and membrane integrity in all TQNPs (except TQNP12.5) were significantly higher than in the CON group. Optimal viability values were detected in TQNP50, followed by TQNP37.5, TQNP25, and TQNP12.5 groups. Notably, there were no significant differences in sperm viability among the CON group and all TQNPs (except TQNP50) (*p* > 0.05). The TQNPs added to the extender had no discernible impact (*p* = 0.07) on the values of sperm morphology (Table 2).

### 3.3. Effects of TQNPs on Kinematic Features of Post-Thawed Buffalo Bull Sperm

The data for kinematic variables of post-thawed buffalo bull sperm are displayed in Table 3. Except for LIN, WOB, and ALH, TQNPs significantly affected all kinematic variables of post-thawed buffalo bull sperm. The TQNP50 group demonstrated the best results for DAP, DCL, VAC, VCL, and BCF, followed by TQNP37.5. TQNP37.5 produced the highest values for STR. Moreover, the TQNP50 and TQNP37.5 groups had greater DSL and VSL values than in other treatments. Similar results were observed in TQNP37.5 and TQNP25 for DAP, DCL, VAP, and VCL. Both total motility and progressive motility had the highest values in TQNP50, while the other groups had similar values of total motility (*p* > 0.05). TQNPs induced a dose-dependent significantly increased PM (*p* < 0.0001). Surprisingly, the addition of TQNPs at a concentration of 12.5 µg/mL had no significant effects compared to the control, as shown in Table 3.

### 3.4. Effects of TQNPs on Acrosome Integrity of Post-Thawed Buffalo Bull Semen

According to Table 4, percentages of viable sperm with intact acrosomes (LSIA) improved significantly (*p* < 0.000) across all treated groups, while percentages of viable sperm with exocytosed acrosomes (LSDA) decreased significantly (*p* < 0.001). In contrast, the addition of TQNPs had no significant effect on the percentages of non-viable sperm with intact acrosomes (DSIA) or exocytosed acrosomes (DSDA) (*p* = 0.30 and 0.020, respectively). In addition, the TQNP12.5 group had higher LSIA values than the CON group (*p* < 0.05) did.

### 3.5. Effects of TQNPs on Total Antioxidant Capacity and Oxidative and Nitrosative Biomarkers in Post-Thawed Buffalo Bull Semen

The data in Table 5 show the effect of different TQNPs on oxidative biomarkers of post-thawed buffalo bull semen. The TQNP50 had higher levels of TAC (*p* < 0.0001) and lower (*p* < 0.004) values of oxidative markers, including MDA and H_2_O_2_, followed by TQNP37.5, but there was no significant difference between the two groups. The levels of MDA and H_2_O_2_ tended to be higher in the CON group, followed by TQNP12.5 and TQNP25 groups without statistical differences. However, no significant difference (*p* = 0.2) was identified among all groups regarding the NO levels. There was a decrease in NO levels by 21.12, 19.7, 8.45, and 4.22% for TQNP50, TQNP37.5, TQNP25, and TQNP12.5, respectively.

### 3.6. Effects of TQNPs on Apoptosis-like Changes

Table 6 displays the effects of the TQNP buffalo extender on apoptosis-like changes. There were significant effects of TQNPs on viable sperm (*p* < 0.0001), with TQNP50 exhibiting the highest viable sperm values, followed by TQNP37.5 > TQNP25 > TQNP12.5. TQNP12.5 had the highest level of early apoptosis (*p* < 0.0001), while the other treated groups showed no statistical evidence (*p* > 0.05). Both groups demonstrated identical results in terms of CON and TQNP12.5 apoptotic sperm (A+/PI+) with similar results. In addition, no significant differences were observed for apoptotic sperm (A+/PI+) among the TQNP37.5, TQNP25, and TQNP50 treatment groups. The percentages of necrotic sperm (A−/PI+) were higher (*p* < 0.0001) in the CON group (*p* < 0.0001) compared with other groups, while the lowest values were noticed in both TQNP50 and TQNP12.5 groups.

### 3.7. Sperm Ultrastructure

**Figure 3:**
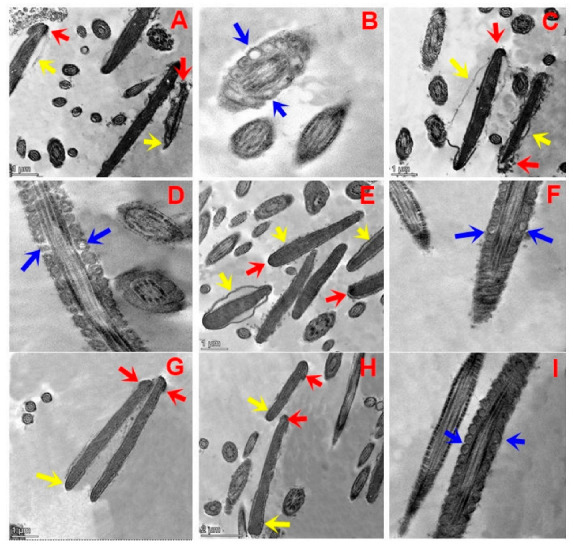
Transmission electron microscopy (TEM) micrographs of post-thawed buffalo sperm in groups treated with 0, 12.5, 25, 37.5, or 50 µg/mL of TQNPs. Control (**A**,**B**) and TQNP12.5 (**C**,**D**) groups exhibited different stages of damage in the sperm nucleus after the freezing–thawing process, whereas (**A**,**C**) indicates that the sperm plasma membrane was swollen and degenerated completely (yellow arrow). In addition, the acrosomal cap was damaged, and acrosomal material diffusion into the formed space was also noticed (red arrow). The longitudinal section of the medial fragments reveals abnormally vacuolated mitochondria in sperm and the absence of crystals ((**B**,**D**); blue arrow) in control and TQNP12.5. In other treated groups, TQNP25 (**E**,**F**), TQNP 37.5 (**G**), and TQNP50 (**H**,**I**), it exhibits numerous sperm cells with normal nucleus and homogenous condensed chromatin surrounded by intact plasma membrane (yellow arrow), and regular acrosome (red arrow). Additionally, mitochondria were regularly placed in longitudinal sections in all treated groups (TQNP25- TQNP50).

Figure 3A–I depict the effects of different concentrations of TQNPs added to a freezing medium on the ultrastructure changes in buffalo sperm after cryopreservation. After a cycle of freezing and thawing, both the control (Figure 3A,B) and TQNP12.5 (Figure 3C,D) groups exhibited distinct phases of impairment, with plasma membrane swelling and complete degeneration (yellow arrow) in buffalo sperm (Figure 3A). In addition, damage in the acrosomal cap, the diffusion of acrosomal dense material into the previously formed space (red arrow), abnormally vacuolated mitochondria, and the absence of crystals (Figure 3B,D; blue arrow) were also noticed in both groups. In other treated groups, TQNP25 (Figure 3E,F), TQNP 37.5 (Figure 3G), and TQNP50 (Figure 3H,I) showed sperm cells with a normal morphology and regular nucleus, homogenous condensed chromatin surrounded by an intact plasma membrane (yellow arrow), and acrosome (red arrow). And mitochondria were regularly placed in all treated groups (TQNP, 25–50).

### 3.8. Effects of TQNPs on Fertility Trial

**Figure 4:**
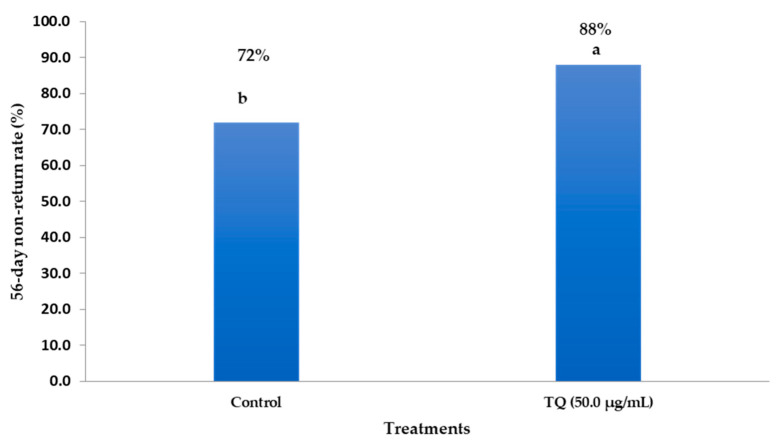
The impacts of inseminated semen supplemented with TQNPs at level of 50 µg/mL on the 56-day non-return rate in buffalo cows. ^a,b^ Values of the same column with different superscripts are significantly different (*p* < 0.05).

As depicted in Figure 4, buffalo cows inseminated with 50 µg/mL of TQNP-enriched freezing medium had higher non-return rates than the control group. The results indicated that 88% (44/50) of cows inseminated with 50 µg/mL of TQNPs exhibited pregnancy rates. In contrast, the control cows inseminated with semen without supplementation showed lower pregnancy rates of 72% (36/50) than those of the TQNP50 group (*p* < 0.05).

## 4. Discussion

Cryopreservation of buffalo semen has noticeable adverse effects on sperm motion, membrane integrity, permeability, and fluidity, which in turn interrupts sperm functionality, viability, mitochondrial functions, and fertilizing ability. Buffalo sperm are susceptible to oxidative stress (OS) induced by cryo-injuries or temperature changes due to their high PUFA contents. Therefore, it is essential to enrich the freezing extenders with potent antioxidants such as TQNPs to prevent the adverse effects of OS caused by cryopreservation. Our data revealed that adding TQNPs to the buffalo sperm freezing extender enhanced sperm parameters (progressive motility, viability, and membrane integrity) and significantly reduced sperm apoptosis and acrosome damage. In addition, TQNPs significantly improved the total antioxidant capacity, decreased oxidative pathways (MDA and H_2_O_2_), and increased the 56-day non-return rate in buffalo cows inseminated with TQNP-cryopreserved sperm. This is the first study to support TQNPs as an additive to the freezing extender, whereas previous research only utilized free TQ to increase the cryo-tolerance of ram sperm [22].

In this research, adding 37.5 or 50 µg/mL of TQNPs to freezing media improved post-thawed buffalo bull semen quality compared to other groups (*p* < 0.05). A previous study showed that adding 1–4% *Nigella sativa* extract to the freezing extender significantly improved the progressive motility in buffalo [15]. Similarly, two studies illustrated that the addition of *Nigella sativa* oil to freezing extender results in a substantial improvement effect in terms of post-thawed semen in ovine [30] and goats [16]. Human studies revealed that *Nigella sativa* seed extract is a practical approach to improve sperm motility [13]. Moreover, low doses of TQ (5–10 µg/mL) can increase sperm motility in culture media in normozoospermic men [45] and in aging mice [46]. In contrast, Iranpour et al. [20] clarified that the high levels of TQ (20 µg/mL) could ultimately impair the sperm motility in culture media. TQNPs are thought to be more effective than free TQ in decreasing OS and supporting mitochondrial function [47]. Mitochondrial respiration as an energy source is key for sperm motility. Numerous studies demonstrated that TQ-loaded liposomes were more effective than free TQ with robust antioxidant activity [21,48]. TQ addition did not affect sperm viability in normozoospermic men [45], while in our work, the addition of TQNPs significantly improved the percentages of sperm viability. High levels of TQ (50 µg/mL) significantly decreased sperm motility and viability by inhibiting sperm mitochondrial function [20]. It has been reported that cryopreservation reduces the membrane integrity of sperm significantly [29]. Therefore, improving the sperm membrane function during cryopreservation is critical for obtaining high fertility outcomes in AI. This could be accomplished by supplementing the freezing extenders with antioxidants that protect the sperm against cryo-damage, such as TQ. In chilled and post-thawed buffalo semen, Awan et al. [15] found that a freezing extender supplemented with *Nigella sativa* extract at 3–5% significantly improved plasma membrane integrity values compared to the stranded group (*p* < 0.05). Additionally, Nasiri et al. [13] found that the *Nigella sativa* extract significantly preserved sperm membrane function of human sperm following cryopreservation. CASA techniques allow the examination of sperm kinetic aspects in real-time, yielding accurate and fast results [49]. They have virtuous predictive significance for fertilization competence [50]. In this research, TQNPs (37.5 or 50 µg/mL) had better results for sperm kinematics than other groups did (CON, TQNP12.5, and TQNP25). Consistent with our findings, the addition of the *Nigella sativa* extract at doses of (1–6%) also enhanced the percentage of progressive motility and other motion parameters (LIN, VCL, VSL, and VAP) (*p* ≤ 0.05) in contrast to the untreated one in cryopreserved human sperm [13]. Moreover, the oral administration of TQ improved sperm kinetics such as VSL, VCL, and VAP in mice exposed to heat stress [18], as evidenced by the improvement in morphology of seminiferous tubes. Therefore, TQ could be considered a natural substance for improving sperm functionality. All affirmative impacts could be clarified by the promising effects of TQNPs on energy production to sperm via affecting aerobic respiration with oxidative phosphorylation singling. The authors believe that these increased sperm motility and velocity characteristics could also be explained by neutralizing OS with TQNPs as compared to the control. According to Miah et al. [30], a considerable improvement in the values of VCL, STR, and ALH of ovine sperm was achieved due to adding 100 µL/mL of *Nigella sativa* oil to the freezing extender. The movement of sperm in the cervical mucus of ewe was positively correlated with the kinetic aspects of sperm, such as VCL [51].

The oxidative stress induced by the cryopreservation process has destructive effects on sperm functionality and structurally. Therefore, oxidative stress in semen should be reduced to preserve sperm functionality and structure during the freeze–thaw cycle [7]. In addition, previous research has confirmed that diminishing the antioxidant capacity and high oxidative stress can disrupt the sperm’s function and structure, such as progressive motility, plasma membrane integrity, and DNA [5], and could increase the percentage of apoptotic sperm [10,52]. This cannot be directly compared with our data. However, we observed that the addition of TQNPs (37.5 or 50 µg/mL) into the freezing extender of buffalo semen resulted in higher values of TAC (*p* < 0.0001) and lower values of MDA (*p* < 0.004) and H_2_O_2_ concentrations (*p* < 0.0001). In contrast, no significant differences were found among the groups in NO concentrations (*p* = 0.02). As evidenced previously, free TQ enhanced the testicular pro-oxidant/antioxidant balance in mice exposed to lead toxicity [31] and diabetes-triggered testicular injury [19]. MDA was significantly reduced, and TQ improved TAC in aging mice [46]. In ram semen [22], TQ significantly reduced cryopreservation-induced TAC, while sperm malformations, DNA damage, and oxidative-related biomarkers (MDA and NO) increased significantly. These potential effects of TQNPs, as observed in this study, result from its antioxidant capacity. Free TQ has been found to positively influence steroidogenesis and spermatogenesis, thus enhancing sexual hormones and resulting in improved reproductive outcomes due to its antioxidant and anti-apoptotic effects [31]. In the freezing trials of buffalo semen [29,53], it has been detected that some nanoparticles can increase antioxidant indices after the freeze–thawing cycle with beneficial effects on sperm functionality. However, many studies have been carried out on adding *Nigella sativa* extract [13,15,16,30]. Still, there are limited data on using TQ in freezing extenders for various animals, such as in ram [22]. Free TQ also has superoxide anion scavenger effects, improved cryotolerance, and indirect antioxidant abilities [15]. Furthermore, the addition of *Nigella sativa* extract to cryopreserved human sperm led to a significant decrease in intracellular ROS production as opposed to that of the control group (*p* ≤ 0.05) [13]. Al-Zahrani et al. [18] found that the oral administration of TQ (5 mg/kg) significantly decreased the oxidative stress and lipid peroxidation induced by HS in mice.

Other oxidative biomarkers (MDA and H_2_O_2_) showed statistically significant changes in response to TQNPs. This characteristic was demonstrated by the ability of free TQ to enhance the mitochondrial function of sperm [12], thus reducing the mitochondrial ROS levels [22]. The motility outcomes found in this trial evidenced the view that TQNP management decreases the levels of mitochondrial oxidative stress. This reflects an improvement in flagellar movement, with TQNPs supplying the energy generated from adenosine triphosphate (ATP) mediated by beta-oxidation [54]. Enhancing the β-oxidation of certain fatty acids at the mitochondrial site with TQ or its nanoform has not been investigated. Additionally, free TQ can stimulate spermatogenesis in rats by preventing mitochondrial dysfunction [19,55]. Moreover, TQ significantly decreased the testicular NF-κB and iNOS, and upregulated the aromatase expression levels in diabetic rats [15].

Acrosome reaction (AR) is a crucial physiological process that sperm must undergo to fertilize an oocyte. However, induced spontaneous acrosome integrity due to cryopreservation reduces fertility [19]. Therefore, it is essential to maintain the acrosome integrity by adding natural molecules to the freezing medium. In this study, TQNPs increased the percentage of intact acrosomes and decreased the percentage of exocytosed acrosomes. Similar to our data, *Nigella sativa* boosts the raw sperm features and acrosome integrity of cyclophosphamide-triggered testis toxicity in mice [17]. It was also found that the *Nigella sativa* extract improved buffalo sperm quality by boosting antioxidant features [15]. Lipid change instigated via oxidative stress is one of the critical aims for higher percentages of sperm cells with an exocytosed acrosome in infertile bulls [7]. The ability of TQNPs to modulate the Ca^2+^ influx into the sperm via the CatSper cation channel demonstrates the protective effect of TQNPs in regulating the acrosome integrity of buffalo sperm [56]. Studies have reported that TQ significantly reduced the apoptotic sperm induced by aging [46], chronic toluene exposure [55], and bleomycin-induced reproductive toxicity [32]. Mitochondrial dysfunction may lead to the induction of apoptosis of sperm cells and reduce the fertilizing capacity. Recently, Shirmard et al. [14] found that TQ provides mitochondrial protection against trastuzumab-induced cardiotoxicity. This increase in mitochondrial membrane potential was probably due to a decrease in in intracellular ROS supported by *Nigella sativa* extract.

Some studies have evidenced that sperm cells exposed to cold shock, such **as** via the cryopreservation procedure, indeed exhibited numerous modifications in their structure after examination through TEM, such as acrosome damage, increased plasma membrane fluidity, and mitochondria dysfunction [5,57,58,59]. In addition, these changes in sperm morphology may induce apoptosis and necrosis-like fluctuations during the cryo-storage and freeze–thaw procedure [58,59].

Our previous research revealed that the freezing medium enriched with some natural antioxidants such as nanocurcumin [25], L-Carnitine [10], lipoic-acid-loaded nanoliposomes [5], and selenium nanoparticles [26] improved the sperm quality and functionality of the cryopreserved sperm in bulls (buffalo and cattle) and rabbit bucks.

In the present study, we found that the supplementation of TQNPs to the extender of buffalo semen could support substantial protection from cryo-damage via supporting the sperm membrane, motion characteristics, and functionality. The current study provides promising results. However, it is necessary to investigate the effects of other nanoparticles as lipid carriers by targeting mitochondrial function during the cryo-storage of buffalo sperm.

In this study, buffalo cows were inseminated with sperm treated with 50 µg/mL of TQNPs to further examine the potential positive effects of this compound on the sperm fertilizing capacity. Based on the in vivo trial, the non-return rate of cows in the TQNP group was 88% compared to 72% in the control group. This confirms that the TQNPs could improve the cryopreserved buffalo semen and contribute to the development of a new type of extender that protects or prevents the adverse effects of the cryopreservation protocol, particularly in species with a high PUFA content in their sperm structure, such as buffalo. The potential of TQNPs to improve the pregnancy rate in buffalo cows is associated with the improvement in sperm motility and reduction in sperm apoptosis and acrosomal damage. Free TQ has anti-oxidative (increasing superoxide dismutase and reducing glutathione enzymes) and antiapoptotic (decreasing the expression of Bax and Caspase-3 in testicular tissues) properties in rats [31]. Various medicinal plants added to the freezing extender can improve the in vivo fertility of buffalo semen, such as *Moringa olifera* leaf extract [8], κ-carrageenan [60], and other antioxidants [11].

## 5. Conclusions

The present study reveals that thymoquinone nanoparticles (TQNPs at doses 25 to 50 µg/mL) improve the quality of buffalo sperm during cryo-storage. For more clarification, the supplementation of TQNPs in extended semen decreases oxidative-related markers (Malondialdehyde and hydrogen peroxide) and enhances the post-thawed sperm kinematic semen parameters (progressive motility, viability, membrane integrity, acrosome integrity, and total antioxidant capacity), indicating an improvement in cryo-tolerance of buffalo bull semen. Moreover, TQNPs (50 µg/mL) improve buffalo cows’ fertility in vivo. Despite these positive effects of TQNPs, additional research is required to clarify other mechanistic pathways, such as metabolites or proteomics studies in buffalo sperm after nanoparticle supplementation into the freezing medium.

## Figures and Tables

**Table 1:** Effect of supplementing Tris-extender with thymoquinone nanoparticles on progressive motility, viability, membrane integrity, and morphology of buffalo bull sperm after equilibration at 5 °C for 4 h.

Treatments ^1^	Sperm Characteristics (%)			
	Progressive Motility	Viability	Membrane Integrity	Sperm Morphology
CON	72.9 ± 1.01 ^c^	74.3 ± 1.36 ^c^	74.3 ± 0.81 ^b^	8.7 ± 0.47 ^a^
TQNP12.5	74.3 ± 1.30 ^bc^	76.1 ± 1.58 ^bc^	74.4 ± 0.95 ^b^	6.3 ± 0.68 ^b^
TQNP25	78.6 ± 1.43 ^ab^	80.1 ± 1.06 ^abc^	80.6 ± 1.17 ^a^	6.6 ± 0.37 ^b^
TQNP37.5	80.0 ± 1.54 ^a^	81.7 ± 1.76 ^ab^	80.3 ± 1.48 ^a^	6.4 ± 0.30 ^b^
TQNP50	80.7 ± 1.30 ^a^	82.4 ± 1.53 ^a^	81.6 ± 1.53 ^a^	7.1 ± 0.55 ^b^
*p* value	0.0004	0.001	<0.0001	0.009

^1^ Thymoquinone nanoparticles (TQNPs) were added to the freezing extender with various levels of 12.5 (TQNP12.5), 25 (TQNP25), 37.5 (TQNP37.5), and 50 (TQNP50) µg/mL, and CON: control group. Sperm morphology is expressed as the percentage of abnormal sperm cells. Results are expressed as Mean ± SE, n = 7. ^a–c^ Values of the same column with different superscripts are significantly different (*p* < 0.05).

**Table 2:** Effect of supplementing Tris-extender with TQNPs on progressive motility, viability, membrane integrity, and morphology of post-thawed buffalo bull sperm.

Extender ^1^	Sperm Characteristics (%)			
	Progressive Motility	Viability	Membrane Integrity	Sperm Morphology
CON	41.4 ± 0.92 ^b^	43.7 ± 0.78 ^b^	44.1 ± 0.86 ^b^	9.7 ± 0.57
TQNP12.5	43.6 ± 0.92 ^ab^	45.6 ± 1.15 ^ab^	44.3 ± 1.32 ^b^	8.4 ± 0.65
TQNP25	47.1 ± 1.84 ^a^	48.0 ± 1.77 ^ab^	49.6 ± 2.20 ^a^	7.9 ± 0.34
TQNP37.5	47.9 ± 1.01 ^a^	48.7 ± 1.23 ^ab^	49.3 ± 1.08 ^a^	8.1 ± 0.46
TQNP50	48.6 ± 1.43 ^a^	49.7 ± 1.34 ^a^	50.3 ± 1.73 ^a^	9.3 ± 0.42
*p* value	0.001	0.02	0.01	0.07

^1^ TQNPs were added to the freezing extender at different concentrations of 12.5 (TQNP12.5), 25 (TQNP25), 37.5 (TQNP37.5), and 50 (TQNP50) µg/mL, and CON: control group. Sperm morphology is expressed as the percentage of abnormal sperm cells. Results are expressed as Mean ± SE, n = 7. ^a,b^ Values of the same column with different superscripts are significantly different (*p* < 0.05).

**Table 3:** Effect of supplementing Tris-extender with TQNPs on kinematic parameters of post-thawed buffalo bull sperm.

Variables (Unit) ^1^	Treatments ^2^					
	CON	TQNP12.5	TQNP25	TQNP37.5	TQNP50	*p* Value
TM (%)	54.5 ± 3.56 ^b^	57.6 ± 1.61 ^b^	57.4 ± 1.47 ^b^	57.6 ± 1.51 ^b^	67.6 ± 1.16 ^a^	0.003
PM (%)	41.9 ± 1.98 ^d^	46.6 ± 1.21 ^cd^	51.4 ± 1.16 ^bc^	53.6 ± 0.93 ^b^	60.4 ± 0.98 ^a^	<0.0001
DAP (µm)	18.1 ± 0.40 ^c^	18.6 ± 0.46 ^c^	19.3 ± 0.50 ^bc^	22.5 ± 1.45 ^ab^	23.6 ± 0.68 ^a^	<0.001
DCL (µm)	28.3 ± 0.68 ^c^	29.2 ± 0.88 ^c^	31.0 ± 0.65 ^bc^	36.4 ± 2.48 ^ab^	37.6 ± 1.27 ^a^	<0.001
DSL (µm)	12.9 ± 0.27 ^b^	13.1 ± 0.56 ^b^	13.6 ± 0.42 ^b^	16.9 ± 1.11 ^a^	16.9 ± 0.49 ^a^	<0.001
VAP (µm/s)	41.0 ± 1.01 ^c^	42.1 ± 1.08 ^c^	44.0 ± 1.35 ^bc^	51.1 ± 3.29 ^ab^	53.5 ± 2.01 ^a^	0.0004
VCL (µm/s)	63.8 ± 1.72 ^c^	65.5 ± 2.05 ^c^	70.4 ± 2.13 ^bc^	82.5 ± 5.64 ^ab^	85.0 ± 3.54 ^a^	<0.001
VSL (µm/s)	29.3 ± 0.69 ^b^	29.8 ± 1.11 ^b^	30.8 ± 1.17 ^b^	38.4 ± 2.50 ^a^	38.2 ± 1.43 ^a^	<0.001
STR (%)	71.0 ± 1.00 ^ab^	70.2 ± 1.74 ^ab^	69.6 ± 0.75 ^b^	74.2 ± 0.58 ^a^	70.8 ± 0.86 ^ab^	0.05
LIN (%)	45.2 ± 0.49	45.2 ± 1.83	43.4 ± 0.68	46.0 ± 1.18	44.6 ± 0.51	0.50
WOB (%)	64.0 ± 0.55	63.6 ± 1.29	62.0 ± 0.84	61.6 ± 1.29	62.6 ± 0.68	0.40
ALH (µm)	2.6 ± 0.13	2.7 ± 0.14	2.7 ± 0.12	2.7 ± 0.16	2.9 ± 0.11	0.70
BCF (Hz)	24.7 ± 0.80 ^abc^	24.0 ± 0.24 ^c^	24.4 ± 0.80 ^bc^	27.3 ± 0.88 ^ab^	27.5 ± 0.54 ^a^	0.003

^1^ TM, Total motility (%); PM, progressive motility (%); DCL, distance curved line (µm); DSL, distance straight line (µm); DAP, distance average path (µm); VAP, velocity average path (µm/s); VCL, velocity curved line (µm/s); VSL, velocity straight line (µm/s); STR, straightness (VSL/VAP; %); LIN, linearity (VSL/VCL; %); WOB, wobble (VAP/VCL; %); BCF, beat cross frequency (Hz); and ALH, amplitude of lateral head displacement (µm). ^2^ TQNPs were added to the freezing extender at different concentrations of 12.5 (TQNP12.5), 25 (TQNP25), 37.5 (TQNP37.5), and 50 (TQNP50) µg/mL, and CON: control group. Results are expressed as Mean ± SE, n = 5. ^a–d^ Values of the same column with different superscripts are significantly different (*p* < 0.05).

**Table 4:** Effects of adding Tris-extender with TQNPs on acrosome integrity of post-thawed buffalo bull semen.

Treatment ^1^	Viable Sperm with Intact Acrosome (LSIA, %)	Viable Sperm with Exocytosed Acrosome (LSDA, %)	Non-Viable Sperm with an Intact Acrosome (DSIA, %)	Non-Viable Sperm with Exocytosed Acrosome (DSDA, %)
CON	33.6 ± 1.08 ^c^	26.2 ± 2.73 ^a^	30.0 ± 2.07	10.2 ± 2.08
TQNP12.5	43.8 ± 0.97 ^b^	16.8 ± 1.36 ^b^	31.4 ± 1.36	8.0 ± 0.71
TQNP25	49.2 ± 0.97 ^a^	16.6 ± 0.51 ^b^	27.2 ± 0.58	7.0 ± 0.71
TQNP37.5	49.4 ± 0.93 ^a^	14.8 ± 0.66 ^b^	29.0 ± 1.30	6.8 ± 0.92
TQNP50	51.4 ± 1.21 ^a^	14.0 ± 0.55 ^b^	28.0 ± 1.52	6.6 ± 0.68
*p* value	<0.0001	<0.0001	0.30	0.20

^1^ TQNPs were added to the freezing extender at different concentrations of 12.5 (TQNP12.5), 25 (TQNP25), 37.5 (TQNP37.5), and 50 (TQNP50) µg/mL, and CON: control group. Results are expressed as Mean ± SE, n = 5. ^a–c^ Values of the same column with different superscripts are significantly different (*p* < 0.05).

**Table 5:** Effect of adding Tris-extender with TQNPs on total antioxidant capacity and oxidative and nitrosative biomarkers in post-thawed buffalo bull semen.

Treatment ^1^	TAC (mM/L)	MDA (nmol/mL)	H_2_O_2_ (mM/L)	NO (µmol/L)
CON	0.21 ± 0.03 ^c^	14.0 ± 0.91 ^a^	0.075 ± 0.004 ^a^	7.1 ± 0.35
TQNP12.5	0.25 ± 0.04 ^c^	13.0 ± 0.89 ^ab^	0.069 ± 0.006 ^a^	6.8 ± 0.54
TQNP25	0.39 ± 0.02 ^b^	10.6 ± 0.91 ^abc^	0.060 ± 0.002 ^ab^	6.5 ± 0.76
TQNP37.5	0.42 ± 0.03 ^ab^	10.2 ± 0.85 ^bc^	0.049 ± 0.004 ^bc^	5.7 ± 0.59
TQNP50	0.54 ± 0.03 ^a^	9.2 ± 0.78 ^c^	0.043 ± 0.003 ^c^	5.6 ± 0.31
*p* value	<0.0001	0.004	<0.0001	0.20

^1^ TQNPs were added to the freezing extender at different concentrations of 12.5 (TQNP12.5), 25 (TQNP25), 37.5 (TQNP37.5), and 50 (TQNP50) µg/mL, and CON: control group. TAC, total antioxidant capacity; MDA, Malondialdehyde; NO, nitric oxide; and H_2_O_2,_ hydrogen peroxide. Results are expressed as Mean ± SE. ^a–c^ Values of the same column with different superscripts are significantly different (*p* < 0.05).

**Table 6:** Effect of adding Tris-extender with TQNPs on sperm apoptotic-like changes of post-thawed buffalo bull sperm (Annexin V/PI assay).

Treatment ^1^	Apoptosis-like Changes (%)			
	Viable (A−/PI−)	Early Apoptotic (A+/PI−)	Apoptotic (A+/PI+)	Necrotic (A−/PI+)
CON	40.4 ± 0.38 ^d^	0.2 ± 0.02 ^c^	38.6 ± 1.07 ^a^	20.9 ± 0.79 ^a^
TQNP12.5	51.8 ± 1.13 ^c^	8.4 ± 0.50 ^a^	39.5 ± 0.67 ^a^	0.4 ± 0.03 ^d^
TQNP25	54.0 ± 1.37 ^c^	3.5 ± 0.85 ^b^	29.4 ± 2.18 ^b^	13.2 ± 1.75 ^b^
TQNP37.5	62.0 ± 0.55 ^b^	1.5 ± 0.00 ^bc^	30.7 ± 0.61 ^b^	5.8 ± 0.06 ^c^
TQNP50	67.4 ± 0.35 ^a^	2.8 ± 0.46 ^b^	29.5 ± 0.44 ^b^	0.3 ± 0.06 ^d^
*p* value	<0.0001	<0.0001	<0.0001	<0.0001

^1^ TQNPs were added to the freezing extender at different concentrations of 12.5 (TQNP12.5), 25 (TQNP25), 37.5 (TQNP37.5), and 50 (TQNP50) µg/mL, and CON: control group. Annexin V/PI assay (Annexin V and propidium iodide (PI) labeling of cells) is a technique applied for distinguishing cell death and recognizing between its various pathways: apoptosis or necrosis. Results are expressed as Mean ± SE. ^a–d^ Values of the same column with different superscript are significantly different (*p* < 0.05).

## Data Availability

Data are available upon request to the corresponding authors.
